# Troponin in diabetic patients with and without chronic coronary artery disease

**DOI:** 10.1186/s12872-015-0051-z

**Published:** 2015-07-21

**Authors:** Carlos Alexandre Wainrober Segre, Whady Hueb, Rosa Maria Rahmi Garcia, Paulo Cury Rezende, Desiderio Favarato, Celia Maria Cassaro Strunz, Marília da Costa Oliveira Sprandel, Alessandra Roggério, Ana Luiza de Oliveira Carvalho, Raul Cavalcante Maranhão, José Antonio Franchini Ramires, Roberto Kalil Filho

**Affiliations:** Department of Clinical Cardiology, Heart Institute (InCor) University of São Paulo, Av. Dr. Eneas de Carvalho Aguiar 44, AB, BL I, Sala 114, Cerqueira César, Sao Paulo, SP 05403-000 Brazil

**Keywords:** Biological markers, Troponin, Diabetes mellitus, Coronary artery disease

## Abstract

**Background:**

Cardiac-specific troponin detected with the new high-sensitivity assays can be chronically elevated in response to cardiovascular comorbidities and confer important prognostic information, in the absence of unstable coronary syndromes. Both diabetes mellitus and coronary artery disease are known predictors of troponin elevation. It is not known whether diabetic patients with coronary artery disease have different levels of troponin compared with diabetic patients with normal coronary arteries. To investigate this question, we determined the concentrations of a level 1 troponin assay in two groups of diabetic patients: those with multivessel coronary artery disease and those with angiographically normal coronary arteries.

**Methods:**

We studied 95 diabetic patients and compared troponin in serum samples from 50 patients with coronary artery disease (mean age = 63.7, 58 % male) with 45 controls with angiographically normal coronary arteries. Brain natriuretic peptide and the oxidative stress biomarkers myeloperoxidase, nitrotyrosine and oxidized LDL were also determined.

**Results:**

Diabetic patients with coronary artery disease had higher levels of troponin than did controls (median values, 12.0 pg/mL (95 % CI:10–16) vs 7.0 pg/mL (95 % CI: 5.9-8.5), respectively; *p* = 0.0001). The area under the ROC curve for the diagnosis of CAD was 0.712 with a sensitivity of 70 % and a specificity of 66 %. Plasma BNP levels and oxidative stress variables (myeloperoxidase, nitrotyrosine, and oxidized LDL) were not different between the two groups. In a multivariate analysis, gender (*p* = 0.04), serum glucose (0.03) and Troponin I (*p* = 0.01) had independent statistical significance.

**Conclusion:**

Troponin elevation is related to the presence of chronic coronary artery disease in diabetic patients with multiple associated cardiovascular risk factors. Troponin may serve as a biomarker in this high-risk population.

**Trial registration:**

http://www.controlled-trials.com Registration number:ISRCTN26970041

## Background

Cardiac-specific troponins (cTn) have received international endorsement as the standard biomarkers for detection of myocardial injury [1]. To enhance diagnostic sensitivity, new methods have been developed recently that can detect very low levels of troponin in the blood and have resulted in improved diagnostic accuracy [2], but have, on the other hand, led to loss of specificity: cTn has become detectable in subjects without acute myocardial injury [3, 4]. Besides having become detectable in subjects without acute heart disease, detectable cTn is associated with cardiovascular comorbidities and higher mortality in the general population [5–8] and in many disease conditions [9, 10].

Assuming that diabetes is a microvascular disease and therefore contributes to chronic myocite injury [11], several mechanisms may contribute to the increased risk of coronary artery disease (CAD) development [12–14], such as metabolic factors (advanced glycation end products and lipoprotein abnormalities) [15], coagulation abnormalities [16], endothelial dysfunction [17] and higher levels of oxidative stress [18]. Consistent with these functional alterations, the presence of type 2 diabetes mellitus was already a known predictor of elevated cardiac troponin in the general population with the older assays [19]. With the new assays, detection in the general population has become more common: circulating high sensitivity troponin T was measurable in 90 % of otherwise healthy diabetic patients [20]. These elevations are related to cardiovascular disease and cardiovascular death in diabetic compared to nondiabetic patients [21].

In patients with chronic CAD, concentrations of high sensitivity troponin T are higher than that in healthy controls [22, 23] and correlate with the incidence of cardiovascular death or heart failure [23, 24] and total mortality [25]. The extent of CAD measured by the coronary plaque burden on cardiac computed tomographic angiography also correlates with high sensitivity troponin T [26].

Therefore, both diabetes mellitus and chronic CAD are known predictors of elevation of cTn [6, 23]. It is not known whether diabetic patients with CAD have different levels of cTn compared with diabetic patients with normal coronary arteries. To investigate this question, we determined the levels of cTn in two groups of diabetic patients: patients with multivessel CAD and those with angiographically normal coronary arteries.

## Methods

### Study subjects

The study included patients with type 2 diabetes mellitus with angiographically documented proximal multivessel coronary stenosis of >70 %, these patients had normal ventricular function and received optimal medical treatment without coronary revascularization. This group of patients with multivessel CAD was compared to patients with type 2 diabetes mellitus and angiographically normal coronary arteries identified by angiogram or coronary tomography, also with normal ventricular function.

The following were considered as exclusion criteria: smoking, left ventricular dysfunction, atrial fibrillation, uncontrolled hypertension (systolic blood pressure greater than 180 mm Hg and/or diastolic blood pressure greater than 100 mm hg), chronic kidney disease (GFR = 45-59 mL/min or less), hepatic impairment, hypothyroidism, recent surgery, and degenerative musculoskeletal disease.

The study conformed to the guidelines set out in the Declaration of Helsinki and was approved by the Ethics Committee of the University of São Paulo Medical School. All participants gave written informed consent for participation in the study.

Patients were instructed to interrupt statin use 45 days before blood collection. Blood samples were collected after a 12-h fast. After centrifugation, plasma was aliquoted and stored at −70 °C until analysis.

### Laboratory analysis

Glucose, glycated hemoglobin (HbA1c), total cholesterol, and triglycerides were determined from serum samples after a 12-h fast, by using specific kits in the automated equipment Dimension RxL (Siemens Healthcare, Newark, USA). HDL-cholesterol was measured by a homogeneous enzymatic colorimetric method specific for Dimension RxL. LDL-cholesterol was estimated using the Friedewald formula.

### Cardiac troponin I

Cardiac troponin-I was determined using the ADVIA Centaur®TnI-Ultra kit (Siemens Healthcare Diagnostics, NY, USA) in the automated equipment of the same manufacturer. The test is an immunoassay that uses a direct chemiluminescence technology and constant amounts of two monoclonal antibodies. An increased TnI concentration is defined as a value exceeding the 99th percentile of a normal reference population. According to the manufacturers, the detection limit is 0.006 ng/mL, the population reference value at the 99th percentile is <0.04 ng/mL, and the coefficient of variation is <10 % at this 99th percentile.

Siemens Centaur ultra is not considered a high-sensitivity assay, instead, it is designated as a contemporary or level 1 assay. The new troponin assays are defined by their ability to detect troponin above the limit of detection in normal individuals and classified according to the percentage of normal individuals detected in four categories: level 1 corresponds to <50 % of measurable normal values, level 2: 50-75 %, level 3: 75-95 %, and level 4: ≥95 %. Only levels two to four are considered high-sensitivity assays [27]. We have used the designation level 1 assay or the general designation: cardiac troponin.

### Brain natriuretic peptide and oxidative and myocardial stress biomarkers

The oxidative stress biomarkers myeloperoxidase and nitrotyrosine were determined using solid-phase enzyme linked immunosorbent assays (Hycult, Uden, The Netherlands). Oxidized LDL was determined using a competitive enzyme linked immunosorbent assay (Mercodia, Uppsala, Sweden).

Brain natriuretic peptide (BNP) concentrations were determined through chemiluminescence immunoassay (Siemens Healthcare Diagnostics, NY, USA).

### Statistical analysis

Data were analyzed using MedCalc (version 12). Median and interquartile ranges were used to show skewed troponin values. The categorical variables are presented as absolute and relative (%) values. A two-tailed *p* value <0.05 was considered significant. Baseline characteristics of patients were examined in coronary artery disease and control groups. The Student’s *t* test was used for continuous variables and the chi-squared test or Fisher’s exact test for categorical variables. The Mann–Whitney test was used for nonparametrical variables. Multivariate analysis was performed using logistic regression, the model being composed of variables with *p* < 0.20 in the univariate analysis.

## Results

Between January 2011 and March 2012, 95 diabetic patients were included in this study. Fifty patients had chronic coronary artery disease while the remaining 45 patients, the control group, had angiographically normal arteries. The clinical and laboratory characteristics of study participants are shown in Table 1. There were no significant differences among the two groups. Patients with CAD had higher concentrations of total cholesterol and LDL cholesterol.

**Table 1:** Patients characteristics

Characteristic^a^	CAD (n = 50)	Controls (n = 45)	*P* Values
Age, mean ± SD, y	63.3 ± 8.3	61.4 ± 9.4	0.34
Female n (%)	20 (42)	26 (60,5)	0.07
Hypertension n (%)	40 (83)	36 (84)	0.42
Waist, mean ± SD (cm)	103 ± 13	108 ± 17	0.09
BMI, mean ± SD, Kg/m^2^	30.6 ± 6.3	32.4 ± 6.7	0.22
Total Cholesterol (mg/ dL)	216 ± 45	193 ± 33	0.01
LDL Cholesterol (mg/dL)	142 ± 41	124 ± 31	0.03
HDL Cholesterol (mg/dL)	37 ± 8	39 ± 8	0.47
Triglycerides (mg/dL)	173 ± 75	163 ± 70	0.55
Serum Glucose (mg/dL)	124 ± 37	134 ± 32	0.18
Hemoglobin A1C(mg/dL)	7.2 ± 1.8	7.3 ± 1.6	0.61
Insulin use n (%)	12 (25)	11 (26)	0.43
Duration of diabetes, y	9.9 ± 9.7	7.07 ± 6.9	0.16

^a^CAD indicates coronary artery disease, BMI indicates body mass index, LDL cholesterol indicates low density lipoprotein, HDL cholesterol indicates High density lipoprotein, Hemoglobin A1C indicates glycated hemoglobin

Plasma BNP concentrations and oxidative stress variables (myeloperoxidase, nitrotyrosine, and oxidized LDL) are shown in Table 2. Concentrations were not significantly different between the two groups.

**Table 2:** BNP and oxidative stress variables

Variable	CAD	Controls	*P* Values
BNP (n = 78), median values (n), pg/mL	37,0 (34)	28.5 (44)	0.08
Nitrotyrosine (n = 68), median values (n), nm/mL	27.9 (36)	28.7 (32)	0.77
Myeloperoxidase (n = 68), median values, ng/mL	33.0 (37)	27.0 (31)	0.96
Oxidized LDL (n = 71), median values (n), mU/L	14.8(35)	11.2(36)	0.09

*BNP* indicates B-type natriuretic peptide, *Oxidized LDL* indicates oxidized low-density lipoprotein

**Fig. 1:**
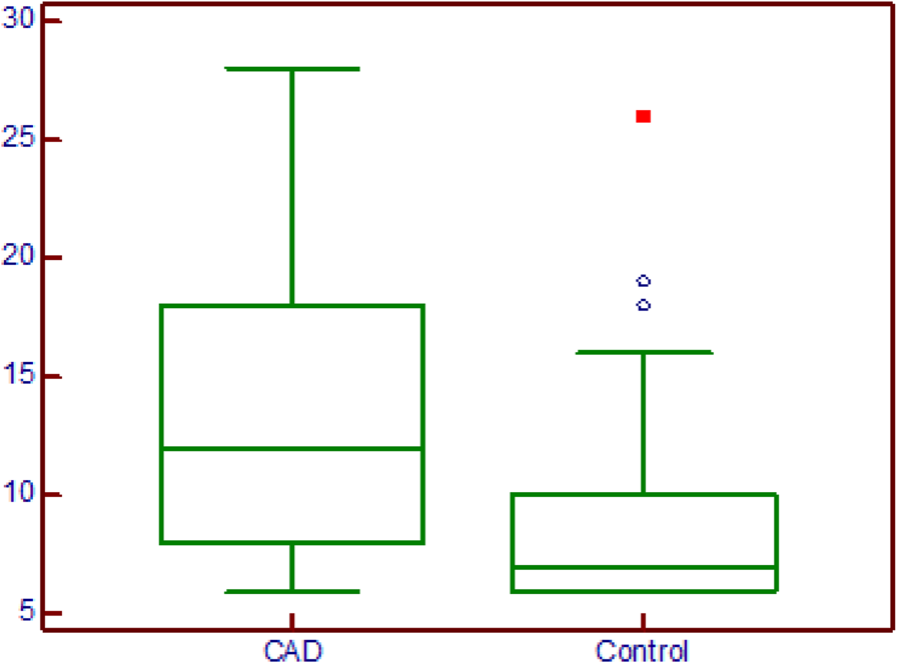
hsTroponinI concentrations in CAD and control groups. Diabetic patients with coronary artery disease had higher levels of troponin than did controls (median values, 12.0 pg/mL (95 % CI:10–16) vs 7.0 pg/mL (95 % CI: 5.9-8.5), respectively; *p* = 0.0001)

**Fig. 2:**
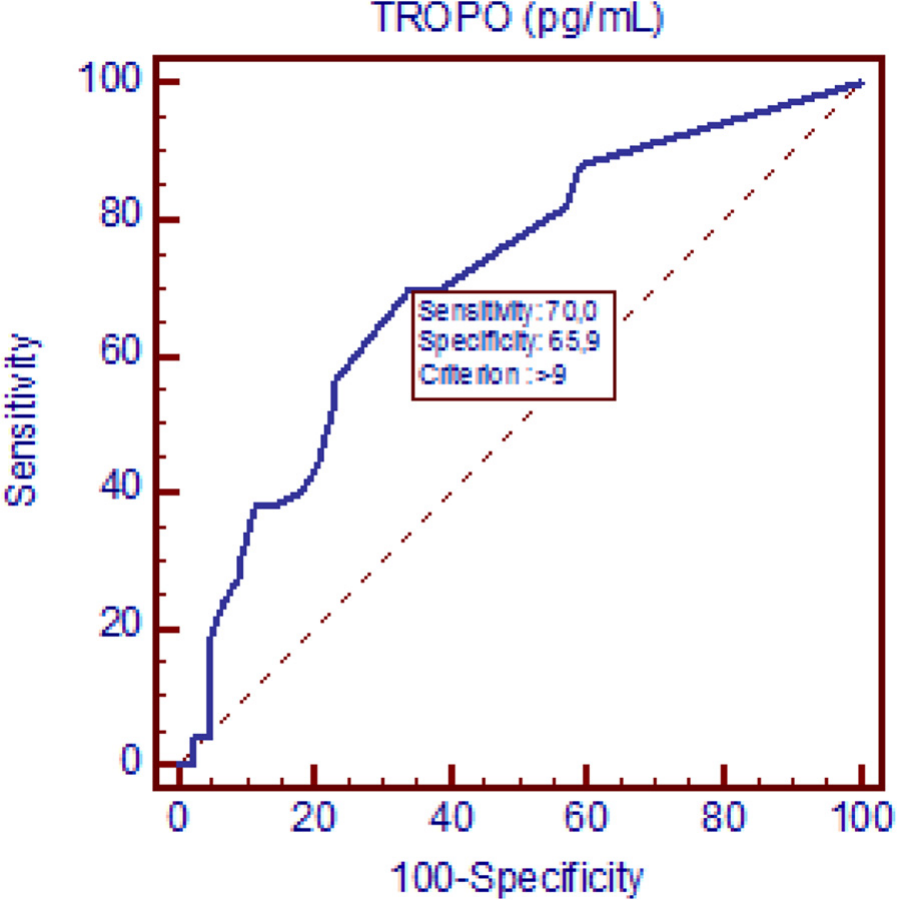
ROC curve for the diagnosis of CAD. The area under the ROC curve for the diagnosis of CAD was 0.712 with a sensitivity of 70 % and a specificity of 66 %

As shown in Fig. 1, diabetic patients with CAD had higher levels of troponin than did controls: median values, 12.0 pg/mL (95 % CI:10–16 pg/mL) vs 7.0 pg/mL (95 % CI: 5.9-8.5 pg/mL), respectively; p = 0.0001. The area under the ROC curve for the diagnosis of CAD, shown in Fig. 2 was 0.712 with a sensitivity of 70 % and a specificity of 66 %. In the multivariate analysis performed and shown in Table 3, gender (p = 0.04), Troponin I (p = 0.01), and serum glucose with a negative correlation coefficient (p = 0.03) had independent statistical significance.

**Table 3:** Multiple regression

Independent variables	Coefficient	Std. Error	r_partial_	t	P
(Constant)	−0.04849				
Waist	−0.002267	0.003964	−0.07906	−0.572	0.5699
Gender	0.2735	0.1304	0.2794	2.098	0.0408
TC	0.004189	0.002269	0.2480	1.846	0.0706
LDL	−0.00007268	0.002797	−0.003604	−0.0260	0.9794
Glucose	−0.003659	0.001730	−0.2815	−2.115	0.0392
Duration_of_diabetes	0.01060	0.008039	0.1799	1.319	0.1930
BNP	−0.001938	0.002445	−0.1092	−0.792	0.4317
Ox_LDL	−0.0003638	0.01383	−0.003649	−0.0263	0.9791
Tropo	0.02919	0.01135	0.3359	2.572	0.0130

## Discussion

In the present study, we evaluated cTn levels in 95 diabetic patients with preserved ventricular function, in the absence of an acute coronary syndrome. We found that concentrations of cTn were significantly higher (p = 0.0001) in diabetic patients with stable coronary artery disease. This finding is consistent with that in other studies that have also shown elevation of cTn in stable coronary artery disease patients [22, 23, 26, 28], but these studies were not specifically designed to test this hypothesis in diabetic patients. The specific contribution of our study is to confirm the association of cTn elevation with CAD in diabetic patients with multiple associated cardiovascular risk factors, demonstrating that cTn is also a useful biomarker in patients with diabetes mellitus. This conclusion is not straightforward, because diabetes mellitus “per se” is associated with an elevation in cTn concentrations [20, 21]. Although a study that investigated high sensitivity Troponin T concentrations in diabetic patients has shown an association with age, gender, and renal function but not with the presence of CAD [20]. In another study that used high sensitivity Troponin I, and a larger number of patients with longer follow-up, troponin I elevation was a predictor of CAD [29]. These two studies did not include multivessel coronary artery disease patients. It is possible that troponin release is related to more diffuse coronary disease, which is a prognostic factor in coronary artery disease [30], and is considered more common among diabetic patients with triple vessel disease [31], which is the group of patients included in our study sample.

Meta-analyses suggest that a previous generation of cardiac troponins I and T had comparable diagnostic and prognostic performance in most clinical settings [32]. The new assays have detected different biological characteristics in high-sensitivity troponin I and T that may be clinically relevant. In a study that compared both biomarkers in a large number of patients, the correlation between the concentrations of the two biomarkers was only of moderate strength. Elevated concentrations of troponin I, but not troponin T, were significantly and independently associated with both prior and the incidence of subsequent acute myocardial infarction [24]. In our study, we have also used troponin I, which again, has shown a good correlation with the presence of CAD. Studies with a larger number of patients comparing the two types of troponin would be necessary to determine which one correlates better with the presence of CAD. Until now, evidence points to the use of troponin I to this purpose.

The reason for the discrepancy between the concentrations of the two types of troponin is not known, and the mechanism that causes troponin release in patients with coronary artery disease is still under investigation. Many hypotheses have been proposed, including transient, clinically silent ischemic episodes and small vessel occlusion, inflammatory processes, cardiomyocyte apoptosis [33], leakage of a cytoplasmatic pool of troponins [34], and plaque microembolization, because the concentrations of high-sensitivity troponin T are related to noncalcified plaque burden and vascular remodeling [28]. In our study, cTn concentrations in diabetic patients without CAD were low and close to the limit of detection of the method used. This result suggests that troponin release is related to the presence of coronary atherosclerosis and not to any other kind of damage to the heart caused by diabetes mellitus.

In the sample studied, total cholesterol and LDL cholesterol concentrations were higher among CAD patients, confirming a finding that is well established among different populations [35]. One unanticipated finding is that serum glucose and hemoglobin A1C concentrations were not different between CAD patients and the control group. However, in the multivariate analysis, serum glucose (with a negative correlation coefficient) was related to CAD in diabetic patients. Hyperglycemia is a risk factor for CAD in epidemiological studies [36]. The unexpected finding of a negative correlation between serum glucose and CAD is probably related to a bias in the study sample, in which the control group had slightly worse, but not statistically significant, glycemic control. Another factor that could explain the lack of difference in glycemic control among CAD and controls is that macrovascular complications do not correlate linearly with HbA1c [37] and the presence of prior vascular disease is one of the predictors of poor cardiovascular outcomes after treatment intensification for insufficient glycemic control [38]. As regards gender, male sex is a well-known risk factor for CAD [39] and also correlated with CAD, in the multivariate analysis, in this study.

### Limitations

The troponin kit used in this study is not among the most sensitive tests available [27], nevertheless, the sensitivity was high enough to show a significant difference between the two groups studied.

Other limitations of this study deserve comment. First, the number of patients is relatively small, and the findings need to be confirmed in larger studies. Second, this is a case–control study that cannot provide information on prognostic implications of subjects with elevated high-sensitivity troponin.

## Conclusion

Troponin elevation is related to the presence of chronic coronary artery disease in diabetic patients with multiple associated cardiovascular risk factors. Troponin may serve as a biomarker in this high-risk population.

## Abbreviations

cTn: Cardiac troponin.
CAD: Coronary artery disease
BMI: Body mass index
LDL cholesterol: Low-density lipoprotein
HDL cholesterol: High-density lipoprotein
TG: Triglycerides
Hemoglobin A1C: Glycated hemoglobin
BNP: B-type natriuretic peptide
Oxidized LDL: Oxidized low-density lipoprotein.
*P* value: Calculated probability of statistical significance
N: Number of patients
GFR: Glomerular Filtration Rate
